# Structure and assembly process of skin fungal communities among bat species in northern China

**DOI:** 10.3389/fmicb.2024.1458258

**Published:** 2024-09-06

**Authors:** Denghui Wang, Fan Wang, Zihao Huang, Aoqiang Li, Wentao Dai, Haixia Leng, Longru Jin, Zhongle Li, Keping Sun, Jiang Feng

**Affiliations:** ^1^College of Life Science, Jilin Agricultural University, Changchun, China; ^2^School of Life Sciences, Central China Normal University, Wuhan, China; ^3^Jilin Provincial Key Laboratory of Animal Resource Conservation and Utilization, Northeast Normal University, Changchun, China; ^4^Jilin Provincial International Cooperation Key Laboratory for Biological Control of Agricultural Pests, Changchun, China

**Keywords:** bats, skin fungal community structure, community assembly, driving processes, environmental fungal reservoir

## Abstract

**Background:**

The skin fungal communities of animals play a crucial role in maintaining host health and defending against pathogens. Because fungal infections can affect the skin microbiota of bats, gaining a comprehensive understanding of the characteristics of healthy bat skin fungal communities and the ecological processes driving them provides valuable insights into the interactions between pathogens and fungi.

**Methods:**

We used Kruskal–Wallis tests and Permutational Multivariate Analysis of Variance (PERMANOVA) to clarify differences in skin fungal community structure among bat species. A Generalized Linear Model (GLM) based on a quasi-Poisson distribution and partial distance-based redundancy analysis (db-RDA) was performed to assess the influence of variables on skin fungal communities. Using community construction models to explore the ecological processes driving fungal community changes, *t*-tests and Wilcoxon tests were used to compare the alpha diversity and species abundance differences between the fungal structure on bat species’ skin and the environmental fungal pool.

**Results:**

We found significant differences in the composition and diversity of skin fungal communities among bat species influenced by temperature, sampling site, and body mass index. Trophic modes and skin fungal community complexity also varied among bat species. Null model and neutral model analysis demonstrated that deterministic processes dominated the assembly of skin fungal communities, with homogeneous selection as the predominant process. Skin fungal communities on bat species were impacted by the environmental fungal reservoir, and actively selected certain amplicon sequence variants (ASVs) from the environmental reservoir to adhere to the skin.

**Conclusion:**

In this study, we revealed the structure and the ecological process driving the skin fungal community across bat species in northern China. Overall, these results broaden our knowledge of skin fungal communities among bat species, which may be beneficial to potential strategies for the protection of bats in China.

## Introduction

The skin fungal communities of bats compete with pathogens for epidermal space and nutritional resources, thereby limiting pathogen growth and contributing to the promotion of host health, reproduction, and development (Rusman et al., 2020; Nguyen and Kalan, 2022). When a pathogen upsets this balance, it impairs the host defense mechanisms and can result in death (Grimaudo et al., 2022). Host, environment and pathogens also affect host skin fungal community structure and composition (Whiting-Fawcett et al., 2024). Hence, it is paramount to elucidate the factors influencing host skin fungal community distribution patterns, as well as the selection pressures driving community assembly.

White-nose syndrome (WNS) is a fungal disease of bats caused by *Pseudogymnoascus destructans* (*Pd*), which primarily invades bat epidermal tissue during hibernation and has led to a dramatic decline in North America (Hoyt et al., 2020). However, bats in Eurasia do not exhibit significant WNS disease symptoms or mortality (Hoyt et al., 2021). It is speculated that bats have shown resistance to *Pd* infection in China (Hoyt et al., 2020), which may potentially be related to the roles of symbiotic skin bacterial communities and their metabolites (Li et al., 2022b). Skin fungal communities are critical in maintaining a host’s microecological balance and health due to their unique ecological niche (Harris-Tryon and Grice, 2022). Previous studies have found that bat skin mycobiomes also influence host resistance to *Pd* infection (Vanderwolf et al., 2021b), and may contribute more to defense against disease than resident bacteria (Martinson et al., 2017). White-nose syndrome restructures bat skin microbiomes (Ange-Stark et al., 2023). Bat skin mycobiomes in the American Southwest vary by host, environment, and feeding behavior (Kearns et al., 2023). The diversity and abundance of skin fungal assemblages on WNS-susceptible bat species is significantly lower than WNS-resistant species (Vanderwolf et al., 2021a). However, the composition and structure of skin fungal communities among bat species in China and the factors that influence them have not been reported.

Fungal community assembly mechanisms and regulatory process have always been a focus of attention in microbial ecology (Li J. et al., 2023). Fungal community assembly may be driven by multiple ecological processes at the same time, with varying relative contributions (Zhu et al., 2024). Neutral theory posits that stochastic processes are crucial to drive microbiomes to convergence or diverge (Coyte et al., 2021), while ecological niche theory suggests deterministic processes such as environmental filtering or selection and species interactions exert more substantial effects (Martinson et al., 2017). Examples of both abound; for instance, fungal pathogen infections alter the selection, spread, and migration processes of amphibian skin microbiota (Wilber et al., 2020), while microbial community assembly in termite mounds is driven by deterministic selection (Chen et al., 2021). Studies have also found that ecological processes varied considerably with host species, diet, and environment (Zhu et al., 2022b). Therefore, quantifying the community assembly processes of bat skin mycobiomes helps to comprehensively understand the structure of fungal communities.

In this study, we selected five widely-distributed bat species across five regions in northern China during the late hibernation period for skin fungal sampling, to clarify the differences in community composition and structure between bat skin and environmental samples, and to explain the processes driving skin fungal community assembly. We had three objectives: (1) to explore the differences in composition, structure, and function of skin fungal communities among bat species in China; (2) to quantify the relative importance of deterministic and stochastic processes in the assembly process of skin fungal communities; and (3) to elucidate the relationship between skin fungal communities and the environmental fungal reservoir, using the species *Rhinolophus ferrumequinum* and *Murina leucogaster*.

## Materials and methods

### Sample collection

We collected skin fungal and environmental samples of five bat species, including *Mu. leucogaster* (*n* = 29, MULE), *Myotis ricketti* (*n* = 15, MYRI), *R. ferrumequinum* (*n* = 64 RHFE), *R. pusillus* (*n* = 15, RHPU), and *R. macrotis* (*n* = 5, RHMA), from sites known as Bat cave (Beijing), Water channel (Henan Province), Temple cave1 (Liaoning Province), Temple cave2 (Shanxi Province), New cave and Gezi cave (Jilin Province). Environmental samples come from Water channel (*n* = 6), Temple cave1 (*n* = 6), New cave (*n* = 5) and Gezi cave (*n* = 8). Data was collected at the end of March and early April 2019 (Table 1). All sample collection processes were approved by the Laboratory Animal Welfare and Ethics Committee of Jilin Agricultural University.

**Table 1:** Bat skin and environmental samples collection information.

Locality (province)	Species	Roosting temperature (mean ± SD)	No. of bats sampled	No. of environments sampled	Sampling date
Temple cave2 (Shanxi)	RHMA	16.5 ± 0.25	5	0	3/18/2019
Water channel (Henan)	RHPU	9.39 ± 0.34	16	0	3/22/2019
Water channel (Henan)	RHFE	10.67 ± 2.53	15	6	3/22/2019
Bat cave (Beijing)	MYRI	8.99 ± 0.97	16	0	3/25/2019
Bat cave (Beijing)	RHFE	10.5 ± 0.14	4	0	3/25/2019
Temple cave1 (Liaoning)	MULE	10.5 ± 0.36	14	0	3/30/2019
Temple cave1 (Liaoning)	RHFE	7.85 ± 0.98	15	5	3/30/2019
New cave (Jilin)	RHFE	7.05 ± 0.21	15	5	4/1/2019
Gezi cave (Jilin)	MULE	7.32 ± 0.52	15	3	4/2/2019
Gezi cave (Jilin)	RHFE	7.41 ± 0.39	15	6	4/2/2019

Based on previous research, we used sterile polyester swabs dipped in sterile water to swab bat forearms and muzzles (5 times) for *Pd* detection (Hoyt et al., 2016). Skin fungal samples were collected using a similar method to obtain skin bacterial samples (Li et al., 2022a). Environmental samples were derived from bat roosting cave walls (∼5 cm) and swabs were wiped back and forth five times. These samples were preserved in 500 μL RNA later (TIANGEN, Beijing, China). The roost temperature of bats was recorded with an infrared thermometer (Fluke, Everett, WA, United States). Then, species and sex were identified, and forearm length and weight were measured for calculating the body mass index (BMI = weight/forearm length). Bats were released in caves immediately after sampling.

### Molecular methods and sequencing

We extracted DNA from swab samples using the Qiagen DNeasy Blood and Tissue Kit (Qiagen, Hilden, Germany), following the manufacturer’s instructions and examined the presence of *Pd* using qPCR. The positive control was *Pd* ATCC MYA-4855 (Ingala et al., 2017). Determined infected (with *Ct* value) and uninfected (without *Ct* value) bat individuals based on the *Ct* values from qPCR results. *Pd* loads were calculated with the formula: *Pd* = 10ˆ [(Ct −22.04942)/−3.34789]. For skin fungal samples, DNA extraction was performed using the E.Z.N.A™ Mag-Bind Soil DNA Kit (OMEGA Bio-tek, Georgia, United States) as described in the instructions. We amplified the ITS1 gene from the ITS1 region using the universal fungal-specific primers ITS1F and ITS2R (Sun et al., 2023). The PCR reaction system and conditions were referenced from a previous research (Kearns et al., 2023). PCR products were purified, quantified, and sequenced on the Illumina MiSeq platform (2 × 300 bp) (Shanghai, China).

We amplified 20,665,935 raw sequences from 130 bat samples [average 158,968 reads/sample (range 34,243–846,865)] and 25 environmental samples with 3,134,019 raw sequences [average 125,360 reads/sample (range 62,472–250,905)]. Sequence analysis was performed using Quantitative Insights Into Microbial Ecology (QIIME) 2 (Estaki et al., 2020). The DADA2 plugin (Callahan et al., 2016) was used to correct errors, quality filtering and chimera removal, along with generation of an amplicon sequence variants (ASVs). Taxonomy was assigned to the sequence features using QIIME2’s feature-classifier classify-sklearn plugin, based on the UNITE fungal database version 8.2^1^.

### Data analysis

We compared *Pd* loads among species using Kruskal–Wallis tests. Skin fungal community composition was calculated for each bat species at the phylum and genus levels. The shared and unique ASVs of the five bat skin mycobiomes were determined by UpSet analysis and the use of Venn Diagrams. In order to clarify differences in skin fungal community structure among bat species, we analyzed alpha diversity indices (Shannon diversity and Observation richness) and tested their differences using Kruskal–Wallis test. A generalized linear model (GLM) based on a quasi-Poisson distribution was used to examined the effects of predictor variables on alpha diversity indices, including sites, BMI, temperature, sex, *Pd* load and *Pd* infection status. Beta diversity was calculated based on Bray–Curtis distance matrix and plotted using non-metric multidimensional scaling (NMDS) with *phyloseq package* (McMurdie and Holmes, 2013). Permutational multivariate analysis of variance (PERMANOVA) was implemented to assess differences in beta diversity among bat species using the *adonis()* function on the *vegan* package (Oksanen et al., 2015). Subsequently, partial distance-based redundancy analysis (db-RDA) was performed to assess the influence of variables (BMI, temperature, sex, *Pd* load, and *Pd* infection status) on skin fungal communities, while controlling for the effects of species and site variables with the *anova.cca()* function of the *vegan* package.

Null model analysis was carried out to quantify the relative contributions of ecological processes to the assembly of skin fungal communities among bat species, including drift, selection and dispersal (Zhang et al., 2022). The beta Nearest Taxon Index (βNTI) was calculated with the *picante* package based on phylogenetic trees (Fouquier et al., 2016) and the relative abundances of ASVs. |βNTI| ≥ 2 indicates that deterministic processes are playing a dominant role in shaping microbial communities, while smaller absolute values (|βNTI| < 2) point to a stronger influence from stochastic processes instead. βNTI was combined with the Bray–Curtis-based Raup-Crick index (RCI) to estimate the important processes shaping skin fungal communities: homogeneous selection (βNTI < −2), heterogeneous selection (βNTI > 2), homogenizing dispersal (RCI < 0.95 and |β NTI| < 2), dispersal limitation (RCI > 0.95 and |βNTI| < 2), and drift (|RCI| < 0.95 and |βNTI| < 2) (Fang et al., 2023). FUNGuild was used to predict the function of bat skin mycobiomes (Nguyen et al., 2016). Through linear discriminant analysis (LDA) size effect (LEfSe) analysis, we determined that skin fungal communities significantly differed among bat species based on LDA > 3.5, *p* < 0.001. Molecular Ecological Network Analysis Pipeline (MENAP) analysis was conducted to understand the structural characteristics of skin fungal communities including complexity (defined as the number of edges and nodes in the network, http://ieg4.rccc.ou.edu/MENA/). A correlation network was constructed based on Spearman correlation (*p* < 0.05, correlation coefficient threshold = 0.5) by selecting the top 30 ASVs at the genus level in terms of relative abundances from the results of the LEfSe analysis^2^ (Gao et al., 2024).

To explore the relationship between the structure of skin fungal communities of bat species and the environmental fungal reservoir, we calculated Shannon diversity and Observation richness, and compared them using *t*-tests and Wilcoxon tests. We then, visualized the beta diversity and performed PERMANOVA to test for significant differences between bat and environment samples. Procrustes analysis was used to assess the consistency in the composition of bat and environment samples using the *Procrustes()* function of the *vegan* package. We also conducted source tracking analysis on bat skin mycobiomes by applying the Fast Expectation–Maximization Microbial Source Tracking (FEAST) method^3^ (Shenhav et al., 2019), using the *FEAST* package to calculate the proportion of host fungal taxa derived from the potential environmental fungal reservoir (Knights et al., 2011). Neutral modeling analysis revealed the role of neutrality and deviations from neutrality in shaping skin fungal communities (Zhu et al., 2022a). The fundamental assumption of the neutral model is that the probability of detecting an ASV on skin is proportional to the relative abundance of that ASV in the environmental reservoir. Calculating the occurrence frequency of ASVs falls within the 95% confidence intervals using the *Hmisc* package (Tong et al., 2019), we categorized ASVs into three groups: neutrally distributed (within the predicted range), over-represented (occurring more frequent than predicted), and under-represented (occurring less frequently than predicted) (Li A. Q. et al., 2023).

## Results

### *Pseudogymnoascus destructans* infection

The *Pd* loads showed no significant differences among the five bat species (Kruskal–Wallis test: Chi-squared = 6.50, *p* = 0.062) (Supplementary Figure S1A). RHFE had the highest *Pd* loads (average *Pd* loads: −4.35) and RHPU had the lowest *Pd* loads (−5.97). Further analysis revealed significant differences in *Pd* loads of RHFE among different sites (Kruskal–Wallis: Chi-squared = 11.16, *p* < 0.05). RHFE had the highest *Pd* loads at Bat cave (−3.29), while the *Pd* loads at Water channel were the lowest (−5.97). Simultaneously, MYRI had the highest prevalence (93.75%), while RHPU was the lowest prevalence (28.57%) (Supplementary Figure S1B). The *Pd* prevalence of RHFE varied across different sites, with the highest prevalence in Bat cave (100.00%), and the lowest prevalence in Temple cave1 (80.00%).

### Taxonomic composition of skin fungal communities in bat species

**Figure 1:**
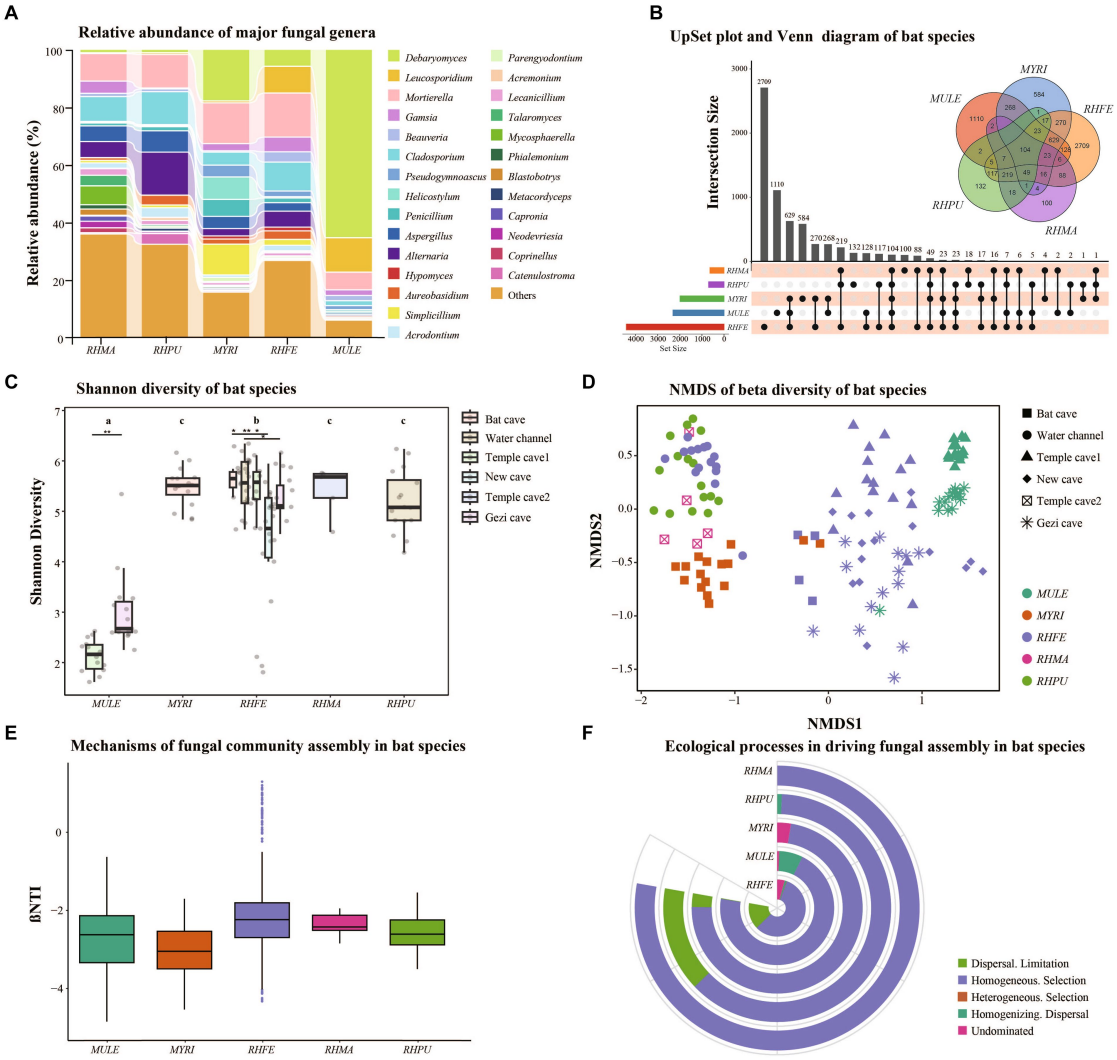
Skin fungal community structure among bat species. **(A)** Alluvial diagram of major skin fungal taxa with relative abundance greater than 0.01 at the genus level. **(B)** UpSet plot and Venn diagram of amplicon sequence variants (ASVs) among bat species. **(C)** Shannon diversity of bat species, with letters and * represent significant differences among groups, *p < 0.05, **p < 0.01. **(D)** Beta diversity of bat species, based on NMDS plot using Bray–Curtis dissimilarity matrices. **(E)** Box plot showing the relative contributions of deterministic processes (|βNTI| ≥ 2) and stochastic processes (|βNTI| < 2) in the assembly of bat species. **(F)** Relative contributions of different ecological processes driving the assembly of bat species.

The skin fungal communities of five bat species were mainly composed of four phyla (Supplementary Figure S1C), of which Ascomycota was the most common. Mortierellomycota and Basidiomycota formed the largest taxonomic phylum in RHFE, while Mucoromycota was the largest in MYRI. At the genus level, there were differences in the relative abundance of the five bat skin fungal communities (Figure 1A). The most common genus in MULE was *Debaryomyces*, while the dominant genus in RHFE was *Mortierella.* The dominant genus in MYRI was *Debaryomyces*, while in RHPU it was *Alternaria*, and in RHMA it was *Mortierella*. Meanwhile, Venn diagram and UpSet analyses revealed that 104 ASVs (5.65%) were shared among bat species (Figure 1B). RHFE possessed the most unique ASVs (2709), followed by MULE (1110), while RHMA had the lowest (100).

### Structure of skin fungal communities across bat species

Shannon diversity (Figure 1C) and Observation richness (Supplementary Figure S1D) were significantly different between bat species (Shannon diversity: Chi-squared = 57.71, *p* < 0.001; Observation richness: Chi-squared = 68.11, *p* < 0.001). Further research clarified significant differences in the alpha diversity of skin fungal communities in RHFE across sites (Shannon diversity: Chi-squared = 65.63, *p* < 0.001; Observation richness: Chi-squared = 73.76, *p* < 0.001). In the GLM analysis, it was found that sites (Shannon diversity: Chi-squared = 413.27, *p* < 0.001; Observation richness: Chi-squared = 83.41, *p* < 0.001) and temperature (Shannon diversity: Chi-squared = 12.24, *p* < 0.001; Observation richness: Chi-squared = 39.20, *p* < 0.001) were the main factors influencing the alpha diversity of skin fungal communities. However, Sex (Shannon diversity: *p* = 0.067; Observation richness: *p* = 0.056), BMI (Shannon diversity: *p* = 0.055; Observation richness: *p* = 0.051), *Pd* load (Shannon diversity: *p* = 0.071; Observation richness: *p* = 0.079) and infection status (Shannon diversity: *p* = 0.086; Observation richness: *p* = 0.071) did not significantly affect alpha diversity.

The NMDS and clustering analyses based on Bray–Curtis dissimilarity (Figure 1D) revealed significant differences in fungal community structure among bat species (NMDS with stress = 0.13; PERMANOVA, Pseudo-F_4, 125_ = 16.53, *p* = 0.001, *R*^2^ = 0.35). Similarly, significant differences were observed in beta diversity among sites (NMDS with stress = 0.14; PERMANOVA, Pseudo-F_5, 124_ = 15.91, *p* = 0.001, *R*^2^ = 0.39). Further investigation showed significant differences in beta diversity of RHFE skin mycobiomes across sites (NMDS with stress = 0.14; PERMANOVA, Pseudo-F_4, 58_ = 8.62, *p* = 0.001, *R*^2^ = 0.37). According to the partial db-RDA analysis, temperature and BMI significantly affected the skin fungal communities (Temperature: Bray–Curtis, PERMANOVA, Pseudo-F_1, 128_ = 17.25, *p* = 0.0001, *R*^2^ = 0.12. BMI: Bray–Curtis, PERMANOVA, Pseudo-F_1, 128_ = 3.18, *p* = 0.0001, *R*^2^ = 0.02), when controlling for the effects of sites and bat species. Sex (*p* = 0.089), *Pd* load (*p* = 0.071), and infection status (*p* = 0.058) did not significantly change the beta diversity of skin fungal communities.

### Deterministic vs. stochastic processes in the assembly of skin fungal communities of bat species

**Figure 2:**
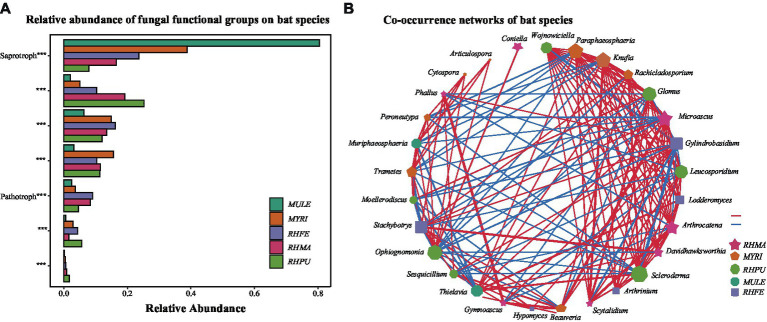
Predicted functional profiles and correlation network of skin fungal communities among bat species. **(A)** Functional classifications of bat species. * indicates significant differences among groups, ***p < 0.001. **(B)** Correlation network of five bat species, lines represent Spearman correlations (*p* < 0.05), with red and blue lines indicating significant negative and positive correlations, respectively, and thicker lines indicating stronger correlations.

Null model analysis indicated that skin fungal community assembly was primarily driven by deterministic processes (|βNTI| > 2), accounting for 87.1% of the ecological processes (Figure 1E). The mechanisms of skin fungal community assembly in bat species were analyzed separately (Figure 1F), and homogeneous selection was the main factor driving community assembly, followed by dispersal limitation and homogenizing dispersal. FUNGuild analysis confirmed the presence of multiple fungal trophic modes in bat skin mycobiomes (Supplementary Figure S1E), which significantly differed among bat species (Figure 2A). Of these, Saprotroph accounted for the largest proportion (16.52%) and was the most abundant in MULE (Kruskal–Wallis: Chi-squared = 87.87, *p* < 0.001); Symbiotroph accounted for the least amount (2.39%) and was highest in RHPU (Chi-squared = 48.82, *p* < 0.001). Concurrently, the trophic modes of the skin fungal community in RHFE across sites were also significantly different, with Pathotroph-Symbiotroph being the most marked (Chi-squared = 36.33, *p* < 0.001).

LEfSe analysis of skin fungal communities well explained differences between species (LDA > 3.5, *p* < 0.001) (Supplementary Figure S2). After MENA analysis, it was found that the skin fungal community structure of RHMA was the most complicated (Total nodes = 210, Total links = 767), followed by MYRI (Total nodes = 162, Total links = 415), and the simplest was RHPU (Total nodes = 28, Total links = 27). Correlation networks showed that some taxa were strongly correlated among bat species (Figure 2B), but their functions were also different (Supplementary Table S1).

### Relationship between skin fungal community structure and environmental fungal reservoir

**Figure 3:**
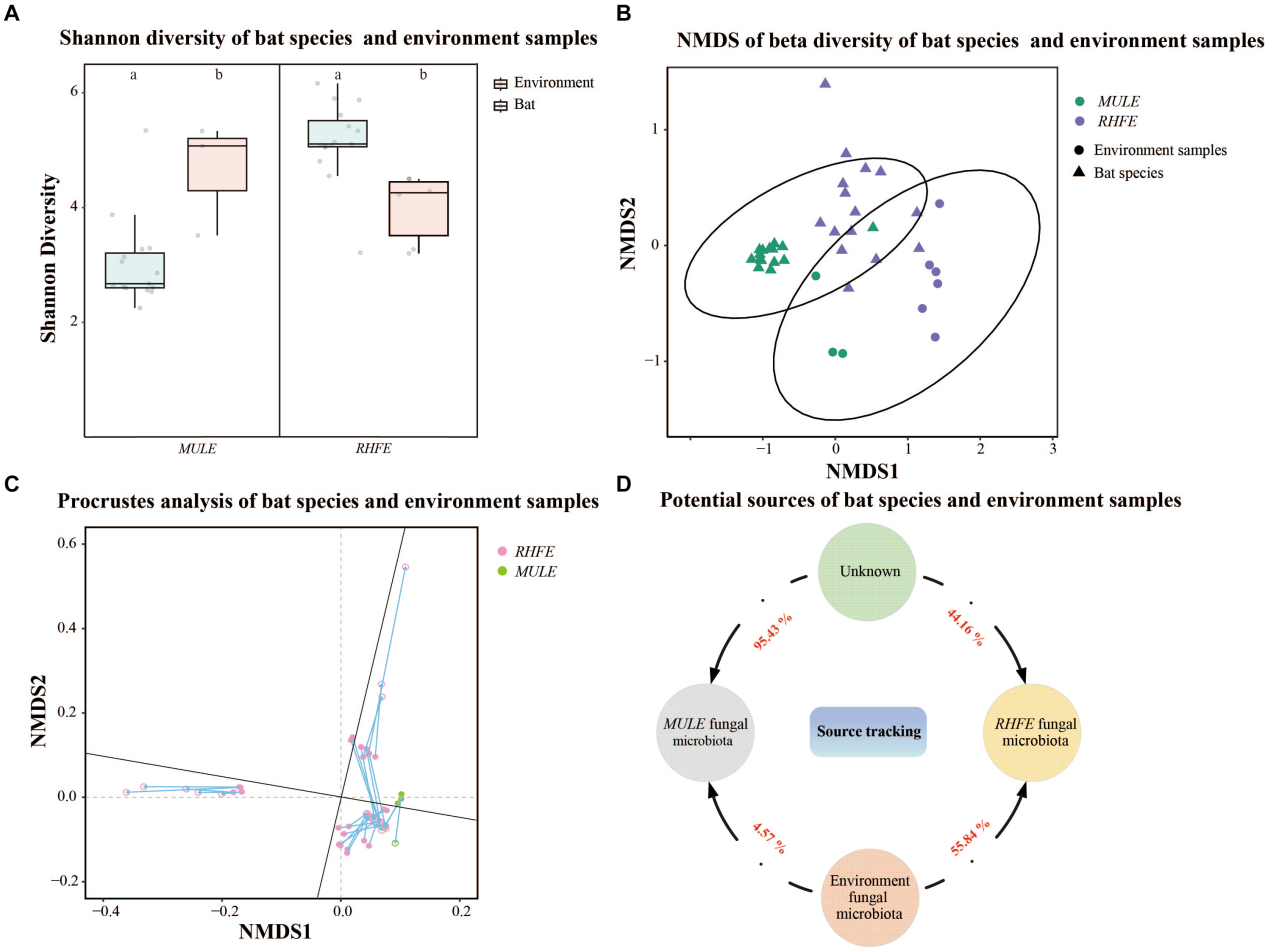
Relationship between bat skin fungal communities and environmental fungal reservoir. **(A)** Shannon diversity of bat species and environment samples. Letters represent significant differences among groups. **(B)** Beta diversity of bat species and environment samples. NMDS plot based on Bray-Curtis distance, with ellipses representing 95% confidence intervals (*CI*). **(C)** Procrustes analysis of bat species and environment samples. Environmental sample points (solid), skin sample points (hollow). **(D)** Potential sources of bat species. Numbers next the arrows indicate the proportion of RHFE and MULE potentially originating from the environmental fungal reservoir and unknown sources.

Shannon diversity and Observation richness of the skin mycobiome in RHFE was higher than in environmental samples (Shannon diversity: *t* = 3.83, *p* = 0.003; Observation richness: *t* = 4.15, *p* = 0.001), while the Shannon diversity and Observation richness of skin mycobiomes in MULE were lower than in environmental samples (Shannon diversity: *W* = 4, *p* = 0.027; Observation richness: *W* = 0, *p* = 0.009) (Figure 3A and Supplementary Figure S1F). Beta diversity indicated that the community structure varied between the environmental fungal reservoir and bat skin mycobiomes (NMDS with stress = 0.10; PERMANOVA, Pseudo-F_1, 117_ = 16.86, *p* = 0.001, *R*^2^ = 0.05; RHFE: PERMANOVA, Pseudo-F_1, 84_ = 7.62, *p* = 0.001, *R*^2^ = 0.05; MULE: PERMANOVA, Pseudo-F_1, 32_ = 15.44, *p* = 0.003, *R*^2^ = 0.34, Figure 3B). Procrustes analysis exhibited good consistency between bat skin mycobiomes and environmental samples (Figure 3C), thus suggesting that the environmental fungal reservoir markedly affected the skin fungal community structure (PROTSET, *M*^2^ = 0.5822, *p* < 0.001).

**Figure 4:**
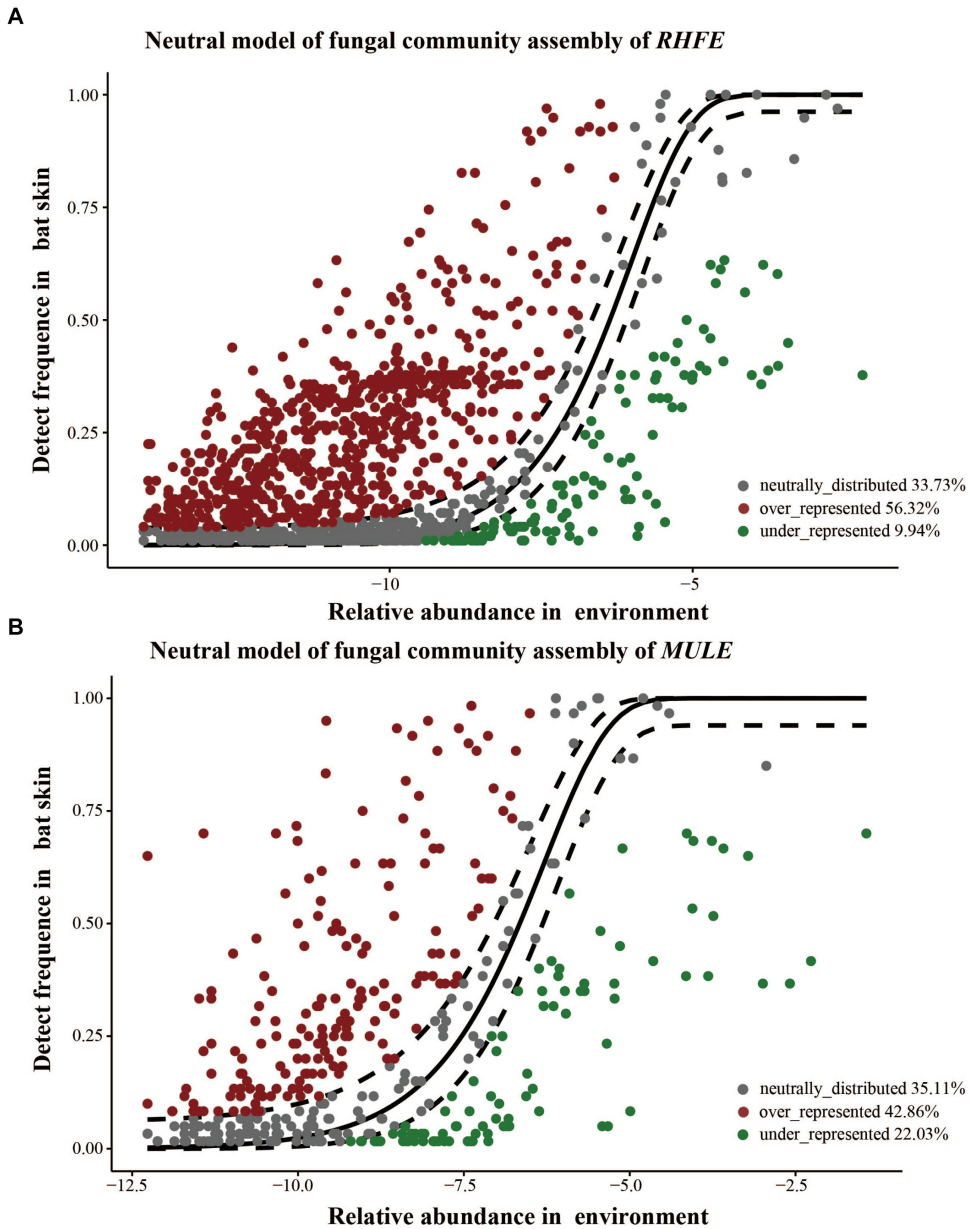
Construction of a neutral model for skin mycobiomes of RHFE **(A)** and MULE **(B)** using the environmental fungal reservoir as the source. ASVs that positively deviating from the model expectations are shown as red dots, those negatively deviated from model expectations are represented as green dots, and ASVs with neutral distribution are shown as gray dots. The solid black line represents the 95% confidence interval around the model prediction.

Source tracking analysis proved that RHFE and MULE acquired 55.54 and 4.57% of available environmental fungi, respectively (Figure 3D). Neutral models explored the shared ASVs between bat skin mycobiomes and environmental samples, which 56.32% (green points) and 9.94% of ASVs in RHFE deviated from neutral predictions (red points in Figure 4A), while 42.86% (green points) and 22.03% (red points in Figure 4B) of ASVs in MULE were subjected to positive and negative selection. Of course, neutral selection also accounted for a certain proportion. In RHFE, there were 905 ASVs that fell into the over-represented groups and 142 ASVs into the under-represented categories. For MULE skin mycobiomes, 177 ASVs were divided into the over-represented groups, and 91 ASVs into the under-represented categories (Table 2).

**Table 2:** Number of over-represented or under-represented ASVs in bat skin fungal communities and the corresponding proportions in the total community.

Species	ASVs status	Total ASVs	Proportion in total community
RHFE	Over-represented	905	42.44% (Gezi cave); 29.14% (Temple cave1); 71.83% (Water channel); 46.71% (New cave)
RHFE	Under-represented	142	10.99% (Gezi cave); 15.14% (Temple cave1); 3.39% (Water channel); 7.21% (New cave)
MULE	Over-represented	177	39.77% (Gezi cave)
MULE	Under-represented	91	18.97% (Gezi cave)

## Discussion

Fungal loads are considered one of the indicators for assessing the transmission risk of WNS, as bats with higher fungal loads exhibit more severe disease symptoms and spread risks (Langwig et al., 2015). This study found no significant difference in *Pd* loads among five bat species in northern China (Supplementary Figure S1A), which was inconsistent with research results from bat species in North America (Frick et al., 2017). In RHFE, there were significant differences in *Pd* loads at different locations (Supplementary Figure S1A), perhaps due to the effect of environmental conditions, seasonal variation, and specific behaviors of bat populations on *Pd* growth (Langwig et al., 2017).

The complexity of fungal community composition can hinder research on microbe-related diseases (Nguyen and Kalan, 2022). These bat skin fungal mycobiomes were mainly composed of five phyla (Supplementary Figure S1B), which are also common in soils worldwide (Schadt and Rosling, 2015), with Ascomycota and Basidiomycota containing mycorrhizal fungi (Peay et al., 2016), and others occupying important ecological niches in animals, including among human, skin fungal communities (Huffnagle and Noverr, 2013). At the genus level, different skin fungal compositions were observed among bat species (Figure 1A), and these *Debaryomyces*, *Leucosporidium*, and *Penicillium* were isolated from the surface of bat skin, with different lifestyles on bat skin. *Debaryomyces* and *Leucosporidium* are symbiotic genera of bat skin, which have the ability to resist pathogen invasion (Vanderwolf et al., 2021a), while *Penicillium* considerably inhibited the growth of *Pd* (Wilson et al., 2017). Only 5.65% of the skin fungal community was shared among the five bat species (Figure 1B), indicating that species-specific fungal communities dominated. However, RHFE had the highest number of both shared and unique ASVs in the skin fungal community composition among bat species, implying that skin fungal diversity was not the sole pathway regulating disease resistance (Mallon et al., 2015), and thus might be associated with key adaptive cohabiting fungal communities (Siomko et al., 2023).

The MENA analysis identified discrepancies in the complexity of skin fungal communities among bat species (Figure 2B). Skin fungal diversity, richness, and interactions affected the number of network nodes and connections, and we found variation in the composition and trophic modes of skin fungal communities among bat species (Supplementary Figure S2 and Figure 2A). Because fungi with different trophic modes exhibit different functions (SupplementaryFigure S1E) (Qin and Han, 2021), their relative composition may be impacted by various factors such as the ecological niche, environmental conditions, and fungal interactions (Vanderwolf et al., 2013). Subsequently, our analysis disclosed that the interaction patterns of skin fungal communities among bat species were distinct (Figure 2B), possibly representing differences in functional niches (Xun et al., 2019). For example, *Arthrinium* and *Stachybotrys* in the skin fungal group of RHFE belong to the Ascomycota and exhibit Pathotroph-Saprotroph, while Phaeosphaeriaceae and *Hypomyces* belong to the Ascomycota and exhibit Saprotroph (Cannon and Kirk, 2007) (Supplementary Table S1). These fungi were involved in organic matter decomposition and nutrient release, especially Phaeosphaeriaceae, which is extremely important to maintaining soil ecosystem function (Wanasinghe and Maharachchikumbura, 2023). Meanwhile, most Ascomycota were saprophytic fungi, with the optimal pH for growth being 7–8 (Yamanaka, 2003). The alkaline pH of skin enhances the virulence of some fungal pathogens (Vylkova, 2017), as demonstrated in *Batrachochytrium dendrobatidis* (Kearns et al., 2017).

Studies of the skin fungal diversity of bat are beneficial for biodiversity conservation and the prevention of pathogen transmission (Langwig et al., 2015). We demonstrated huge differences in both alpha diversity (Figure 1C and Supplementary Figure S1D) and beta diversity (Figure 1D) of skin fungal communities, which may be attributed to habitat conditions and ecological characteristics (Vanderwolf et al., 2015). The simultaneous discovery revealed that both site and temperature drove variation in the diversity and structure of skin fungal communities is similar to findings from a study of bat skin bacterial communities (Grisnik et al., 2020). The invasion of fungal pathogens can lead to a decrease in the richness and diversity of host skin microbiota (Allender et al., 2018). However, in our study the *Pd* load and infection status had no significant effect on the skin fungal diversity of bat species, consistent with other previous research (Vanderwolf et al., 2016). The impact of *Pd* on the skin microbiota of bat species varied. For example, the presence of *Pd* did not affect the richness or evenness of fungal taxonomic units in *Eptesicus fuscus* or *M. lucifugus*, but affected the evenness in *P. subflavus*, with *Pd*-positive bats having a dramatically higher evenness (Ange-Stark et al., 2023). Once again, species and environment were important factors shaping the skin fungal community.

The assembly processes of skin fungal communities are crucial for understanding the adaptability of skin microbiota to environmental changes in ecosystems (Zhou and Ning, 2017). We found that deterministic processes, particularly homogeneous selection, were the main mechanisms regulating the assembly of bat skin mycobiomes (Figure 1E), similar to the assembly processes of microbial communities in agricultural soils and termite mounds in eastern China (Jiao et al., 2020). High levels of homogeneous selection may be influenced by ecological habits, interspecies interactions, and environmental factors, leading to convergence in the composition of skin fungal communities (Fodelianakis et al., 2022). The assembly mechanisms of skin fungal communities also varied among bat species (Figure 1F). For example, in the assembly of skin fungal communities in RHFE, five distinct driving processes were identified, with four showing stochasticity, accounting for 49.55%. This suggests that the assembly mechanisms of skin fungal communities are strongly effected by host selective pressures (Adair and Douglas, 2017), such as, pathogen attack (Holz et al., 2018) and skin physiological characteristics (including pH, temperature, and humidity) (Chen et al., 2018). Additionally, this study found that stochastic processes (homogenizing dispersal, undominated processes, and dispersal limitation) explain a small proportion of skin fungal community diversity across bat species (Figure 1F), highlighting the synergistic effect of multiple assembly process (Gao et al., 2020). Although stochastic processes were essential in the assembly of bat skin fungal communities, deterministic processes remained the primary driving force in assembly.

Environmental factors also influenced skin microbial community structure (Skowron et al., 2021). Procrustes analysis identified a significant correlation between bat skin mycobiomes and adjacent environmental samples (Figure 3C). However, the dramatic differences in the diversity and structure of skin fungal communities among bat species compared to the environmental fungal reservoir (Figures 3A,B), illustrates how the assembly of skin fungal communities could not be entirely mapped onto environmental fungal communities but were filtered by abiotic conditions, species, and diseases (Ange-Stark et al., 2023). Source tracking analysis confirmed that the proportion of skin fungi originating from the environmental fungal reservoir is not high (Figure 3D). Likewise, for many environmental fungal taxa, these characteristics make them more suitable for free-living in environmental media, resulting in low levels of dispersal and selection on host skin (Kearns et al., 2017).

Ecological processes also played a vital role in driving the assembly of skin fungal communities (Li J. et al., 2023). Neutral models illustrated the differences in ecological processes driving the assembly of bat skin mycobiomes (Figure 4) compared to skin bacterial communities (Li A. Q. et al., 2023). Deviations from neutral processes drove the composition of skin fungal communities on bat species, with similar results found in the assembly of microbial communities in diseased lungs (Venkataraman et al., 2015), possibly on account of differences in dormancy strategies between bacteria and fungi, thereby affecting the breadth and composition of community ecology (Chen et al., 2021). ASVs deviating from the neutral model distribution may be well-adapted to hosts or selected by hosts and other environmental pressures (Tong et al., 2019), with the moisture and nutrients of the skin surface potentially favoring the growth of certain microorganisms (Grice and Segre, 2011). Simultaneously, a higher proportion of over-represented fungal taxa (Table 2) suggest that the host could autonomously select beneficial fungi to attach to the skin surface, thereby resisting the adverse effects of the external environment (Chen et al., 2018). For example, haze exposure disturbs the skin fungal community, which promotes the growth of *Talaromyces* and deviates from neutral assembly process (Yan et al., 2023). Certainly, there were some potential limitations to consider in the interpretation of our study results. For instance, the fit of this neutral model was constrained by assumptions about ecological characteristics, as well as temporal and spatial scales (Morris et al., 2013). Moreover, there remained a considerable amount of unexplained variation in the residual partition, which could attribute to undetected environmental variables and species assemblages. However, the results of both the null and neutral models supported the robustness of our conclusions.

## Conclusion

Our research findings showed significant differences in the structure, composition, and trophic modes of skin fungal communities among bat species. Habitat and temperature in particular were significant drivers of changes in the skin fungal community. Using null model analysis to examine the deterministic processes driving the assembly of skin fungal communities, we found evidence for significant differences in the assembly mechanisms among bat species. The diversity and structure of bat skin fungal communities was remarkably different from the environmental fungal reservoir, with the neutral model implying that deviation from neutral processes was the primary driver for the structure of skin fungal communities in RHFE and MULE. These results led to a deeper understanding of structure and quantify the relative importance of assembly processes of skin fungal communities among bat species, enrich the study of skin mycobiomes, understand the interrelationships between host-environment-pathogen-fungal community dynamics, and provide a scientific basis for protecting the health of bat populations.

## Data Availability

All raw sequence data are deposited in the NCBI sequence read archive with accession no. PRJNA1086123.

## Footnotes

^1^ https://doi.org/10.15156/BIO/786380035

^2^ https://www.bioincloud.tech

^3^ https://github.com/cozygene/FEAST
