# Supplementary material for: Structure and assembly process of skin fungal communities among bat species in northern China
Source: Front Microbiol. 2024 Sep 6;15:1458258. doi: 10.3389/fmicb.2024.1458258 (PMC11414763; doi:10.3389/fmicb.2024.1458258)
Supplement: Supplementary file 1 [file Data_Sheet_1.PDF]

Structure and Assembly Process of Skin Fungal Communities Among Bat Species in Northern China

Denghui Wang [1], Fan Wang [1], Zihao Huang [1], Aoqiang Li [2], Wentao Dai [3], Haixia Leng [3], Longru Jin [3], Zhongle Li [1,4,*], Keping Sun [3,*], Jiang Feng [1,4]

[1] College of Life Science, Jilin Agricultural University, Changchun 130118, China.

[2] School of Life Sciences, Central China Normal University, Wuhan 430079, China.

[3] Jilin Provincial Key Laboratory of Animal Resource Conservation and Utilization, Northeast Normal University, Changchun 130117, China.

[4] Jilin Provincial International Cooperation Key Laboratory for Biological Control of Agricultural Pests, Changchun 130118, China.

* Correspondence to: lzy1514316@126.com and sunkp129@nenu.edu.cn

Supplementary material

Table S1 Functional predictive statistical table of skin fungal community at the genus level among bat species.

Species	Taxon	Trophic Mode	Guild
MULE	*Thielavia*	Saprotroph	Dung Saprotroph-Plant Saprotroph-Wood Saprotroph
MULE	*Muriphaeosphaeria*	NA	NA
MYRI	*Paraphaeosphaeria*	Pathotroph-Saprotroph	Fungal Parasite-Plant Pathogen-Plant Saprotroph
MYRI	*Knufia*	Pathotroph	Animal Pathogen
MYRI	*Rachicladosporium*	NA	NA
MYRI	*Beauveria*	Pathotroph-Saprotroph-Symbiotroph	Endophyte-Lichen Parasite-Plant Pathogen-Undefined Saprotroph
MYRI	*Trametes*	Saprotroph	Undefined Saprotroph
MYRI	*Peroneutypa*	Pathotroph-Saprotroph	Plant Pathogen-Plant Saprotroph
MYRI	*Cytospora*	NA	NA
MYRI	*Articulospora*	NA	NA
RHFE	*Arthrinium*	Pathotroph-Saprotroph	Plant Pathogen-Wood Saprotroph
RHFE	*Hypomyces*	Saprotroph	Litter Saprotroph-Soil Saprotroph-Wood Saprotroph
RHFE	*Stachybotrys*	Pathotroph-Saprotroph	Fungal Parasite-Plant Pathogen-Plant Saprotroph
RHFE	*Cylindrobasidium*	NA	NA
RHFE	*Lodderomyces*	NA	NA
RHMA	*Microascus*	Pathotroph-Symbiotroph	Endophyte-Plant Pathogen
RHMA	*Arthrocatena*	Saprotroph	Fungal Parasite-Wood Saprotroph
RHMA	*Davidhawksworthia*	NA	NA
RHMA	*Scytalidium*	Saprotroph-Symbiotroph	Endophyte-Litter Saprotroph-Soil Saprotroph-Undefined Saprotroph
RHMA	*Gymnoascus*	Saprotroph	Soil Saprotroph-Undefined Saprotroph
RHMA	*Phallus*	Saprotroph	Dung Saprotroph-Undefined Saprotroph-Wood Saprotroph
RHMA	*Coniella*	Saprotroph	Undefined Saprotroph
RHPU	*Wojnowiciella*	Pathotroph-Saprotroph-Symbiotroph	Animal Pathogen-Endophyte-Plant Pathogen-Undefined Saprotroph
RHPU	*Glomus*	Epiphyte	Epiphyte
RHPU	*Leucosporidium*	Pathotroph-Saprotroph	Plant Pathogen-Wood Saprotroph
RHPU	*Scleroderma*	NA	NA
RHPU	*Sesquicillium*	Symbiotroph	Ectomycorrhizal
RHPU	*Ophiognomonia*	NA	NA
RHPU	*Moellerodiscus*	Pathotroph-Saprotroph	Animal Pathogen-Plant Pathogen-Undefined Saprotroph

Figure S1 The *Pd* infection status and prevalence, composition, structure and trophic mode of skin fungal communities among bat species, and the alpha diversity of bat skin mycobiome and environmental samples. (a) Box plot of *Pd* infection intensity among bat species. * denotes significant differences among groups, *P < 0.05. (b) Bar graph of *Pd* infection prevalence among bat species. (c) Alluvial diagram of the relative abundances of skin fungal taxa (phylum level) among bat species. (d) Observation richness of skin fungal communities among bat species, with letters and * indicating significant differences among groups, *P < 0.05, **P < 0.01, ***P < 0.001. (e) Bar graph of trophic mode of skin fungal communities among bat species. (f) Observation richness of bat skin mycobiome and environmental samples, with letters representing significant differences between groups.

Figure S2 Cladogram of ASVs among bat species, based on linear discriminant analysis (LDA) score > 3.5, P < 0.001, with letters representing the position of ASVs in the figure.
