# Supplementary figures and images for: Structure and assembly process of skin fungal communities among bat species in northern China
Source: Front Microbiol. 2024 Sep 6;15:1458258. doi: 10.3389/fmicb.2024.1458258 (PMC11414763; doi:10.3389/fmicb.2024.1458258)

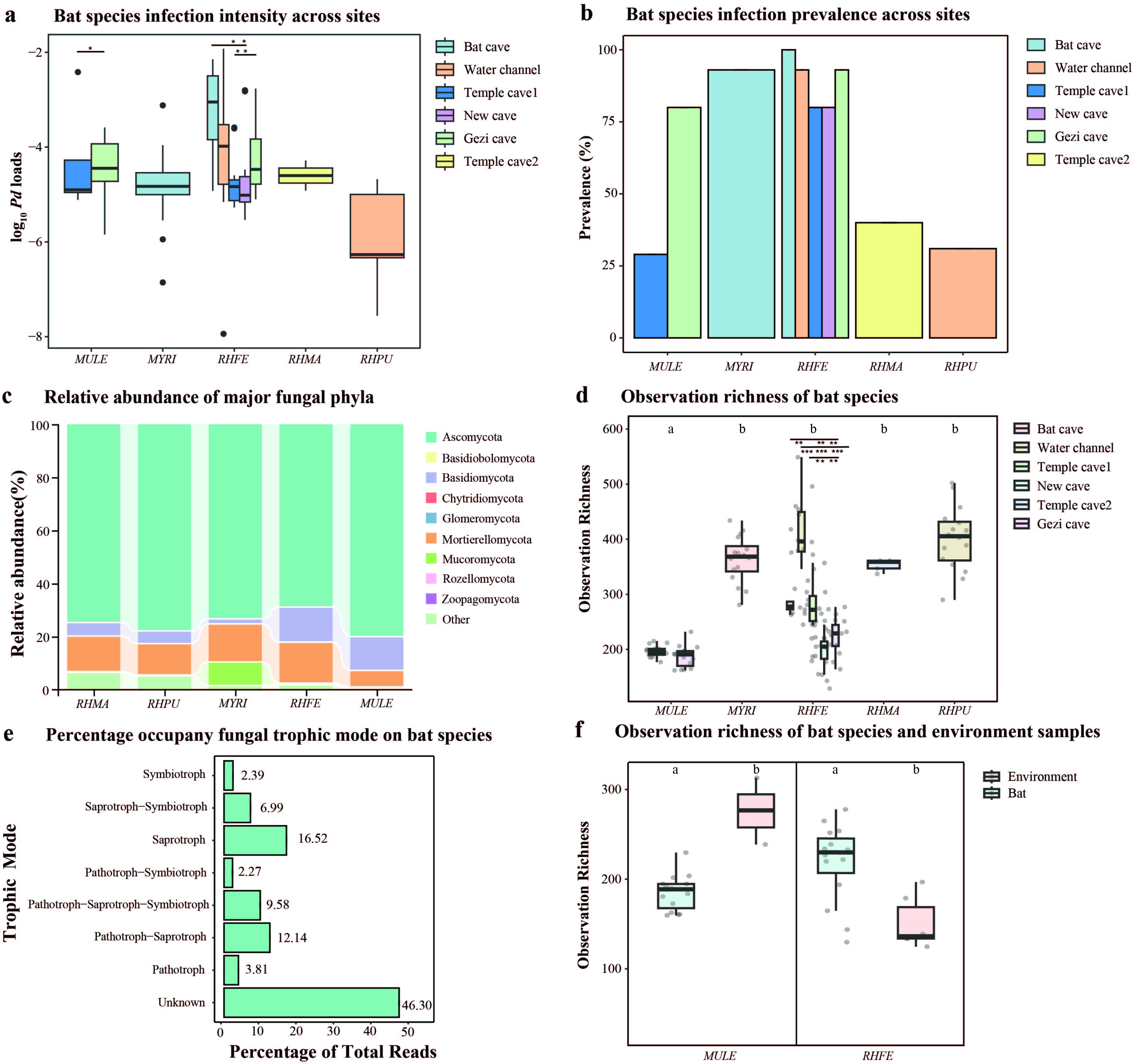

Supplement: Supplementary file 2 [file Image_1.jpeg]

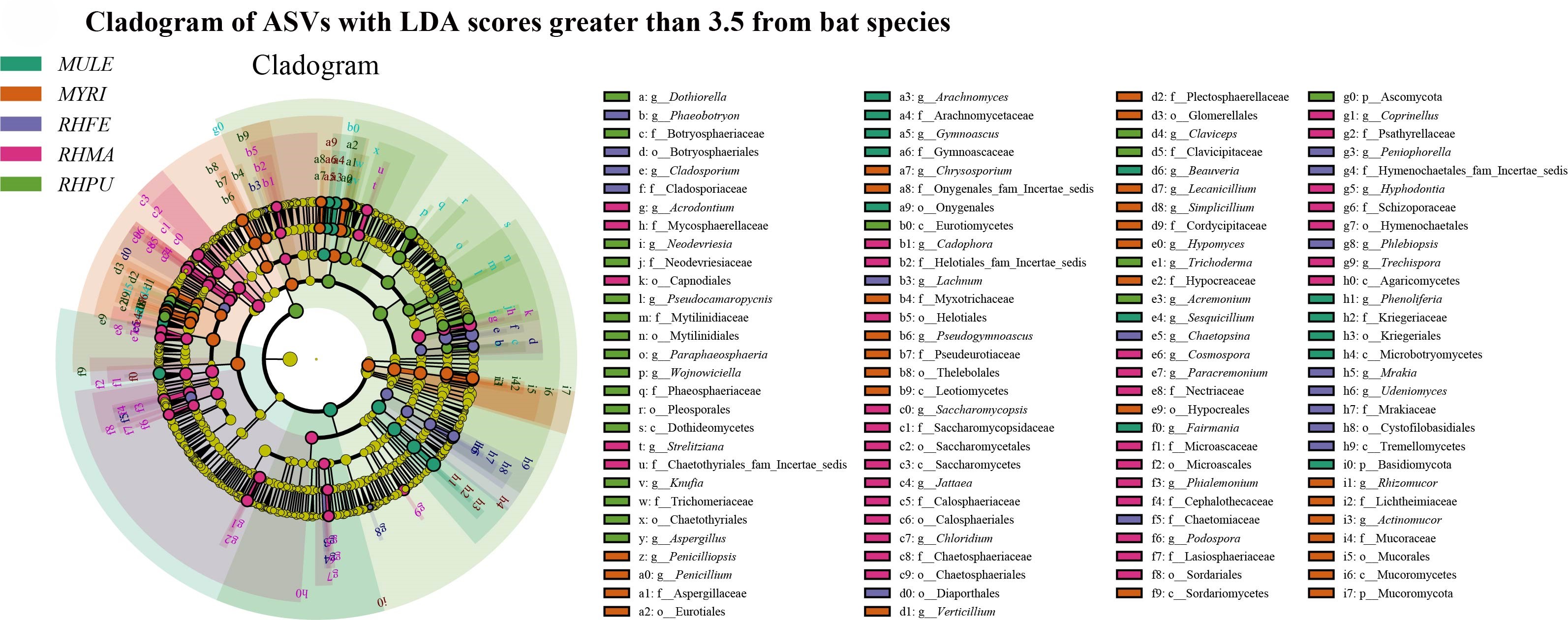

Supplement: Supplementary file 3 [file Image_2.jpeg]
